# Side effects of adjuvant chemotherapy and their impact on outcome in elderly breast cancer patients: a cohort study

**DOI:** 10.2144/fsoa-2020-0076

**Published:** 2020-08-10

**Authors:** Valentina Zanuso, Vittorio Fregoni, Lorenzo Gervaso

**Affiliations:** 1 Humanitas Cancer Center, IRCCS Istituto Clinico Humanitas, 20089 Rozzano (MI), Italy; 2 Humanitas University, 20090 Pieve Emanuele (MI), Italy; 3 ASST Valtellina e Alto Lario, UOC Medicina Generale, 23035 Sondalo (SO), Italy; 4 European Institute of Oncology (IEO) IRCCS, Gatrointestinal Medical Oncology and Neuroendocrine Tumors, 20141 Milan, Italy; 5 Molecular Medicine Program, University of Pavia, 27100 Pavia, Italy

**Keywords:** adjuvant chemotherapy, breast cancer, cardiotoxicity, dose reduction, elderly

## Abstract

**Aim:**

Breast cancer patients over the age of 65 are more likely to suffer chemotherapy side effects, with premature discontinuation, which negatively affects survival.

**Methods:**

We conducted a retrospective cohort study enrolling breast cancer patients; dose reductions or interruptions of chemotherapy have been collected, as well as side effects. Progression-free survival was determined by Kaplan–Meier and evaluated for its association with reduction/suspension. The study included 128 women (median age: 71).

**Results:**

Nineteen patients experienced cardiotoxicity, while dosage of chemotherapy was reduced in 23 patients (18.0%), and 14 (10.9%) had premature interruptions. Dose reduction/interruptions were associated with numerically worse progression-free survival (78.2 vs 94.8 months; p = 0.10).

**Conclusion:**

Reduction/discontinuation of chemotherapy due to side effects affected nearly 30% of our population, potentially worsening outcomes.

## Lay abstract

Breast cancer patients over the age of 65 are more likely to suffer chemotherapy side effects, which negatively affect survival. We conducted a retrospective study of 128 elderly breast cancer patients, collecting changes in chemotherapy doses and schedules, as well side effects. Progression-free survival (PFS) was calculated and evaluated for its association with reduction/suspension. Nineteen patients experienced cardiotoxicity, while dosage of chemotherapy was reduced in 23 patients (18.0%), and 14 (10.9%) had premature interruptions. Dose reduction/interruption were associated with numerically worse PFS (78.2 vs 94.8 months; p = 0.10). Occurrence of reduction/discontinuation of chemotherapy and cardiotoxicity in this population could potentially worsen outcomes.

Breast cancer is the most commonly diagnosed cancer in women, and it is a leading cause of cancer-related mortality worldwide [1]. Age is the major risk factor for the development of the tumor [2] and around 40% of breast cancers are diagnosed in over 65-year old patients. Compared with younger women, older women are more likely to have breast cancer with estrogen receptor and progesterone receptor expression, with or without HER2 overexpression [3]. HER2-positive tumors decrease from 22% among women younger than 40 years to 10% in women 70 years or older [4], whereas tumor size and nodal involvement increase with age [3,5]; this may be partly explained by delayed diagnosis in older women. Considering immunohistochemical and morphological features, high-risk breast cancer will benefit from the addition of systemic adjuvant chemotherapy [6], but the same disease in older women is not always managed properly according to guidelines [7]. This population has, generally, more co-morbidities, worse performance status and is more likely to suffer chemotherapy side effects compared with young patients. The higher rate of toxicities, including cardiologic toxicity straight related to chemoimmunotherapy, often leads clinicians to reduce doses, delay schedules or even prematurely interrupt treatments, which negatively affect survival of these patients [8]. However, literature reports show that older women who are in good health can tolerate chemotherapy similarly to younger patients [9], and the more severe profile of toxicity in these patients has not meaningfully affected the benefits of adjuvant chemotherapy [10,11].

Cardiologic toxicity represents a potential adverse event, acute or chronic, of many antineoplastic agents [12]. This is particularly relevant for anthracyclines and anti-HER2 agents [13,14], the main drugs for the treatment of breast cancer, which could lead to symptomatic or asymptomatic left ventricular disfunction [15]. Current European guidelines define cardiotoxicity as reduction greater than 10% in ejection fraction (EF) without symptoms or greater than 5% when associated with clinical manifestation, below 55% threshold [12]. This is a common and wide definition, applied broadly to cancer patients [16], while no specific suggestions are provided for elderly subjects, which often present co-morbidities and already reduced cardiac function. In this setting, studies have been performed testing biochemical biomarkers [17,18] in order to detect early, asymptomatic, cardiac disfunction and schedule proper clinical controls [19,20]. The aim of this study was to evaluate the rate of main toxicities in this specific setting, especially focusing on cardiotoxicity, the rate of discontinuation and eventually their correlation with outcomes.

## Methods

We conducted a retrospective cohort study enrolling consecutive outsetting breast cancer patients treated at the Medical Oncology Unit of Maugeri hospital, University of Pavia from 01/2010 to 12/17. Inclusion criteria were female sex, age over 65 and indication to adjuvant chemotherapy after multidisciplinary tumor board evaluation. The study protocol was approved by hospital Institutional Review Board. Regimens and adherence to chemotherapy, intended as dose reductions or early interruptions have been collected for each patient by review of electronic medical records, as well side effects (cardiologic, hematologic, gastrointestinal [GI] and any ≥G2 adverse event) occurred during the treatment period. Cardiotoxicity was defined as a reduction greater than 10% in EF without symptoms or greater than 5% when associated with clinical manifestation, below 55% threshold. Rates of hematologic and cardiologic toxicity and rate of chemotherapy dose reduction/discontinuation have been calculated. Progression-free survival (PFS) were performed by Kaplan–Meier and evaluated for its association with chemotherapy reduction/suspension and toxicities occurrence using log-rank test.

## Results

### Study population

We identified 128 patients who were over 65 years old and received adjuvant chemotherapy for breast cancer during the study period. All of those were included in our study population. The median age at diagnosis was 71 years (range: 65–81). Patients characteristics are outlined in Table 1. Thirty-two patients (25.0%) in this cohort did not have any co-morbidities, including arterial hypertension, obesity, diabetes and cardiologic abnormalities, 45 (35.2%) had one disease associated, 33 patients (25.8%) two diseases and 18 (14.1%) more than two significant co-morbidities. Specifically, in our population 21 patients (16.4%) had an altered glucidic homeostasis at the time of diagnosis, with impaired glucose tolerance in six cases (4.7%) and overt diabetes in 15 (11.7%).

**Table 1.** Patient characteristics (n = 128).

Characteristic	Number (%)
**Age (years)**	
– 65–70	57 (44.5)
– 70–75	45 (35.2)
– >75	26 (20.3)
**Co-morbidities, n**	
– 0	32 (25.0)
– 1	45 (35.2)
– 2	33 (25.8)
– >2	18 (14.1)
**Metabolic disorders at baseline**	
– None	107 (83.6)
– Impaired glucose tolerance	6 (4.7)
– Diabetes	15 (11.7)
**Histology**	
– Ductal	116 (90.6)
– Lobular	10 (7.8)
– Other	2 (1.6)
**Immunohistochemical features**	
– Luminal A	23 (18.0)
– Luminal B	26 (20.3)
– HER2 positive	42 (32.8)
– Triple negative	37 (28.9)
**Chemotherapy regimens**	
– Antracyclines and taxanes (sequential)	61 (47.7)
– Antracyclines only	33 (25.8)
– Taxanes only	23 (18.0)
– No antracyclines or taxanes	11 (8.6)
– Anti-HER2 agents	42 (32.8)
**Dose reduction of chemotherapy**	
– No	105 (82.0)
– Yes	23 (18.0)
**Early discontinuation of chemotherapy**	
– No	114 (90.1)
– Yes	14 (10.9)
**Toxicities during chemotherapy (≥G2)**	
– Leukopenia/neutropenia	27 (21.1)
– Febrile neutropenia	5 (3.9)
– Nausea/vomiting	32 (25.0)
– Diarrhea	9 (7.1)
– Mucositis	11 (8.6)
– VTE	9 (7.1)
**Recurrence**	
– No	110 (85.9)
– Yes	18 (*14.1*)

VTE: Venous thromboembolism.

The overwhelming prevalent histology was ductal carcinoma (90.6%); 49 patients had luminal subtypes (38.3%), 42 (32.8%) had HER2-positive tumors and 37 women (28.9%) presented with a triple-negative breast cancer. Luminal A subtypes received chemotherapy if nodal involvement was present (>3 positive lymph nodes). The most used chemotherapy regimen was sequential anthracyclines plus taxanes (61/128, 47.7%) followed by anthracyclines monotherapy in 33 patients (25.8%) and taxanes monotherapy in 22 (17.2%). Forty-two women (32.8%) received trastuzumab, according to tumor biology. In this cohort, at time of completion of data collection, 18 patients (14.1%) experienced recurrence (local or distant).

### Dose reduction/interruption of treatment

Dosage of chemotherapy, compared with calculated full dose, was reduced in 23 patients (18.0%); 14 cases due to hematologic toxicity (60.9%), while seven patients had chemotherapy reduced since first cycle for co-morbidities (30.4%). Moreover, 14 out the total (10.9%) prematurely interrupted the adjuvant treatment. Leukoneutropenia was the most common cause (5/14, 35.7%), followed by allergic reaction to paclitaxel (three case, 21.4%) and cardiologic toxicity (two, 14.3%). In the setting, patients over 75 were the most likely to interrupt treatment (6/14, 42.8%).

**Figure 1.**
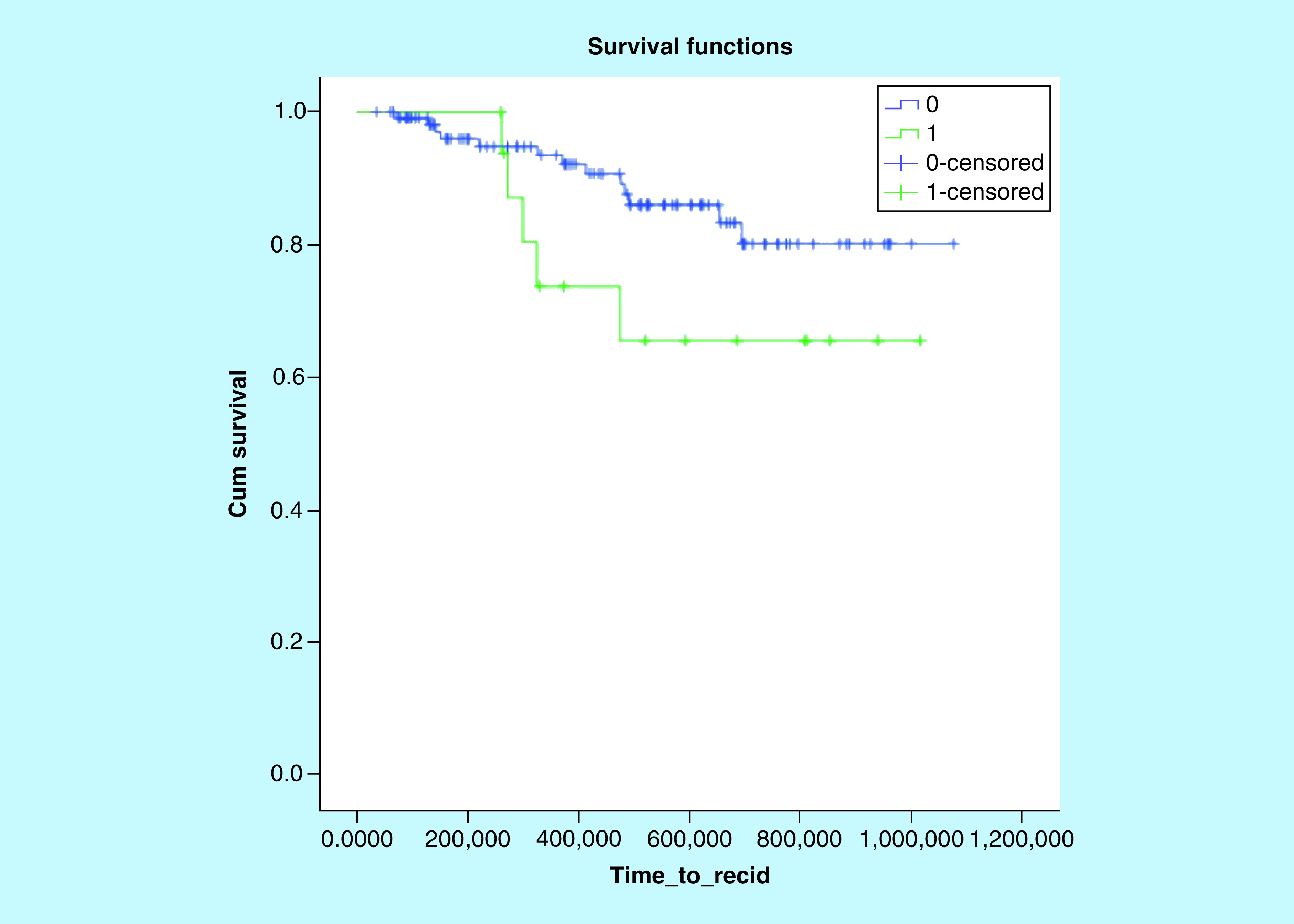
Kaplan–Meier curve for progression-free survival and chemotherapy reduction/discontinuation.

Median PFS in the whole cohort was 92 months (95% CI: 86.6–99.1). Excluding patients who received upfront reduced chemotherapy, dose reduction or premature interruption of the treatment for any reasons were associated with numerically worse PFS, even though this was not statistically significant (78.2 months [95% CI: 61.2–95.1] vs 94.8 months [95% CI: 88.4–101.3]; p = 0.10; Figure 1).

### Cardiotoxicity

**Figure 2.**
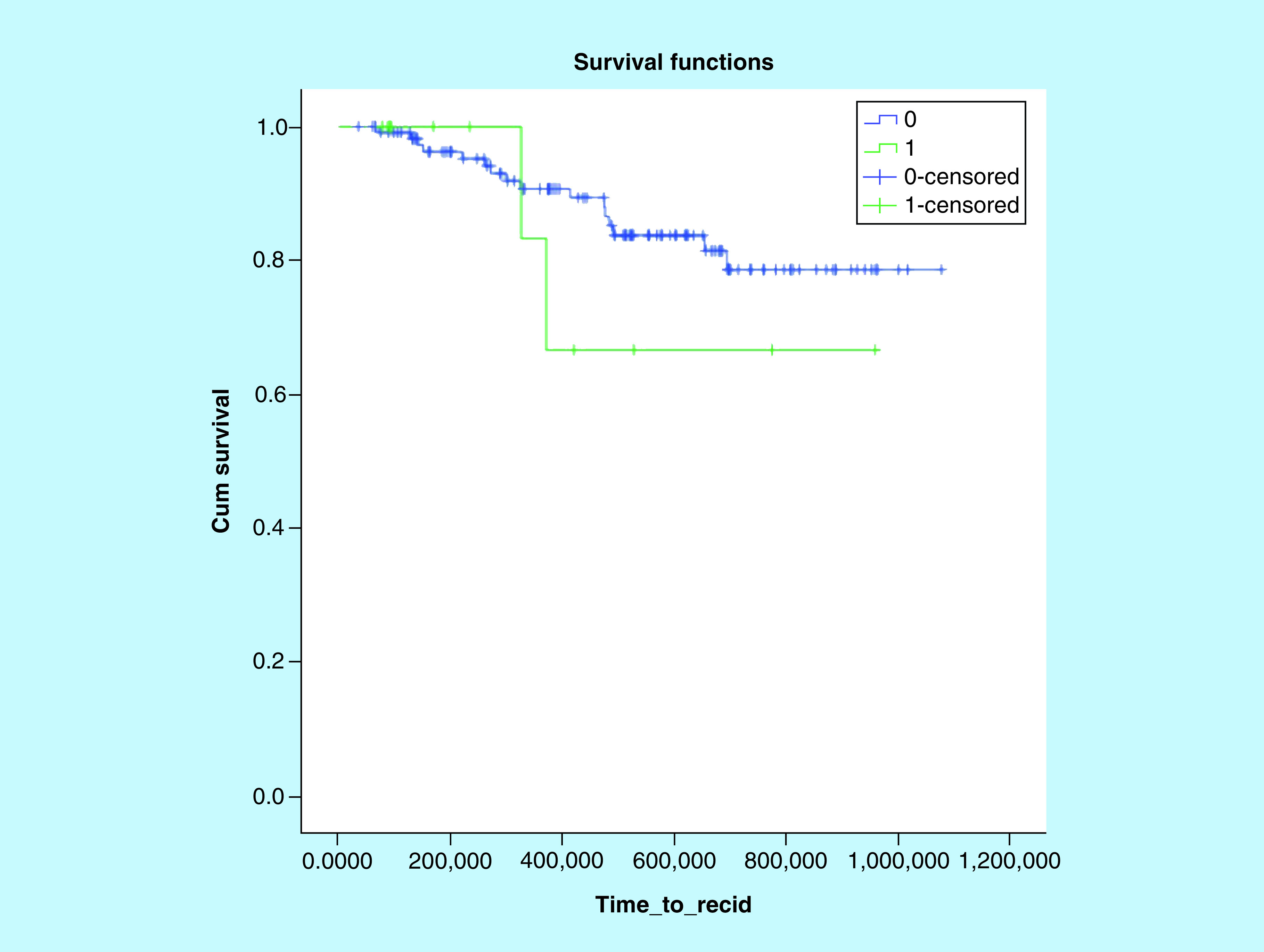
Kaplan–Meier curve for progression-free survival and cardiotoxicity.

In our cohort, 19 patients experienced cardiologic toxicity (14.8 %) during a median follow-up of 44 months (5–99 months). Details are shown in Table 2. After chemotherapy, 12 patients had a reduction in left ventricular ejection fraction, asymptomatic in 11 cases (8.6%) and detected by scheduled echocardiography, while only one patient (0.8%) presented with tachyarrhythmia. Almost all patients (10/12, 83.3%) had significant co-morbidities and seven of those (58.3%) more than two. Similarly, cardiotoxicity occurred in seven women, six without any symptoms associated (4.7%) and one patient (0.8%) with thoracic pain and dyspnea. Overall, eight out 19 events (42.1%) were in women between 70 and 75 years old, while only five in the elder slot (>75 years old), possibly reflecting a closer medical monitoring in these patients. Patients who experienced cardiologic toxicity had worst PFS, but not statistically significant (75.6 months [95% CI: 52.5–98.6] vs 93.5 months [95% CI: 87.1–99.9], p = 0.41; Figure 2).

**Table 2.** Cardiologic side effects during adjuvant treatment.

Drug	Type of event	Number (%)
Chemotherapy	Symptomatic EF reduction (≥5% from baseline)	1 (0.8%)
	Asymptomatic EF reduction (≥10% from baseline)	11 (8.6%)
Trastuzumab	Symptomatic EF reduction (≥5% from baseline)	1 (0.8%)
	Asymptomatic EF reduction (≥10% from baseline)	6 (4.7%)
**Total**		19 (14.8%)

EF: Ejection fraction.

### Other toxicities

Excluding cardiotoxicity, in our dataset 84 patients (65.6%) had at least one adverse event ≥G2. As expected, the most common was GI toxicity: one quarter of the total (n = 32) experienced nausea or vomiting, requiring outsetting medical therapy. Hematologic toxicity, represented by leukopenia and neutropenia, was common in our population and affected 27 patients (21.1%), while five cases (3.9%) had febrile neutropenia and required hospitalization for the management. Granulocyte-colony stimulating factors were not used in prophylaxis but administrated after the first event of neutropenia G4 ore febrile neutropenia. Nine patients presented venous thromboembolism during adjuvant chemotherapy (7.1%), higher than expected. Alterations in glucidic metabolism occurred in 35 cases who were negative at baseline (35/107, 32.7%): 17 patients developed impaired glucose tolerance (15.9%) and 18 had a full diagnosis of diabetes (16.8%) starting therapies and controls.

## Discussion

We performed a retrospective cohort trial evaluating overall tolerability of adjuvant chemotherapy in elderly breast cancer patients and found an incidence of cardiologic toxicity of 14.8% during a median follow-up of 44 months, which is similar to previous reports in the same population [20,21]. On the other hand, 18.0% reduced the dose of chemotherapy and 10.9% early interrupted the treatment; these rates are considerably lower compared with other studies [22,23]. Patients who started chemotherapy at a lower dose from the first cycle were included in the population, since we wanted to assess specifically the impact of dose reduction/discontinuation in the whole population. Even though long follow-up is required to observe differences in survival in adjuvant setting, we found a trend toward worse PFS in patients who experienced cardiotoxicity or did not receive the full adjuvant treatment.

Age is one of the main risk factors for breast cancer and it represents a trend topic in oncology: overall global survival is increasing, and a significant number of breast cancer happened in women aged over 65 years old [2]. This led to the necessity to reconsider treatment paradigms for those patients, often excluded from clinical trials and undertreated for fear of severe/fatal adverse events.

Adjuvant chemotherapy is the cornerstone for treatment for high-risk breast cancer, in particular for large tumor sizes or diffuse nodal involvement. Terrific advantages and progress have been done in this field, starting from the idea of occult micrometastasis [24] up to tailoring therapy according to molecular profile in order to minimize side effects and avoid useless treatments [25]. However, there still is some uncertainty about the management in older women which are not always treated according to current guidelines and such lapses can adversely affect survival. In our study, 10% of the total prematurely interrupted the chemotherapy due to bad tolerance and sides effects. This rate is consistent to one reported by Klepin *et al.* [22], while dose reduction in our study are considerably lower (18 vs 51%), partially because we did not account for patients who directly started chemotherapy at a lower dose. Moreover, accordingly to this study, we did not find a statistical correlation between co-morbidities and severe adverse events G3 or higher due to chemotherapy. This result is in contrast with previous retrospective trials [23,26], probably due to the exclusion of some common conditions (e.g., arterial hypertension, osteoporosis and arthritis). To the present date, few studies have investigated this topic: in a recent systemic analysis [27], only three studies out of 34 analyzed the presence of co-morbidities and their role on tolerability of chemotherapy. Concerning outcome, with limitations due to the retrospective nature and low number, patients who did not receive full adjuvant course of chemotherapy had higher rate of relapse (25.8 vs 21.6%) and showed a trend to worse PFS. In our opinion, due to growing attention to the elderly population, more investigations are required to better clarify this aspect.

After secondary malignancies, cardiovascular disease is the main cause of morbidity and mortality among breast cancer survivors [28]. We, therefore, focused on cardiotoxicity, which is frequently a cause of chemotherapy omission. A study of anthracycline use in fit elderly patients showed an increased risk of cardiac dysfunction [21], while another showed an age-related effect [29]. Long-term rate of congestive heart failure after chemotherapy was 19% for anthracyclines-based regimens and 14% for nonanthracycline ones. Due to retrospective design of the study, we cannot provide a long-term evaluation of cardiac function after chemotherapy, so we primarily analyzed acute events: around 10% of the patients receiving anthracyclines experienced a decrease in left ventricular function, which was mainly asymptomatic. Our results are consistent with literature reports: Lotrionte *et al.* [30] in their metanalysis showed that symptomatic events happened in 6% of cases, climbing up to 18% with asymptomatic ones; the same events due to trastuzumab affected 5.5% of the total, with only one symptomatic patient. On the whole, cardiotoxicity occurred in 15% of the cohort: this result is clearly higher than the ESMO guidelines rate, which considers the total population undergoing adjuvant chemotherapy [12]. From our experience, it is clear, once again, how cardiotoxicity is mainly asymptomatic in early phases: many studies addressed this question trying to identify early cardiac disfunction. Cardinale *et al.* [31] recently showed that the cardiac damage usually happened within the first year of treatment, depending on dosage and EF at baseline. The authors also proposed a new definition for cardiac disfunction: EF reduced by 10% or EF absolute value ≤50%; by this definition, they identified 9.7% of cases, but since no stratification by age was performed [31], it is unclear the applicability in our population. Considering outcome, as expected, PFS was worse in patients who experienced left ventricular disfunction, but this was not statistically significant. According to the current state of the art, it appears urgent to establish common pathways from definition of the toxicity to surveillance and, moreover, biomarkers able to detect early cardiac disfunction before it becomes clinically evident.

Then, we focused on other adverse events that usually complicate chemotherapy: the most common one in our dataset was hematologic toxicity. Leukopenia and neutropenia ≥G2, in particular, happened in one patient out four and five of them presented febrile neutropenia and required hospitalization, potentially worsening outcome in elderly patients. Our results are in line with the CALGB 49907 and CALGB 361004 studies [22], where the same adverse event occurred in 28% of the cohort. Muss *et al.* addressed the same question in their study [10] and found a similar rate of febrile neutropenia (6%) in the over 65-year old population. Since granulocyte-colony stimulating factors were not used in prophylaxis, the relative lower percentage of events in this setting could be attributable to a closer clinical monitoring. GI adverse events, including nausea/vomiting, diarrhea and constipation, have been collected and evaluated as well. We included also G2 toxicities, which are generally less considered in randomized control trials compared with G3–G4 but, however, could be relevant in frail patients. For this reason, in our cohort we observed nausea/vomiting in around a fourth of the total, and diarrhea in 7%, higher than rates reported in the literature [10,32] where only grade ≥3 have been reported. On the other side, we had fewer G3–G4 toxicities, less than 5% accounting for GI adverse events, reflecting that a timely and early detection of these events can avoid treatment interruptions and hospitalizations.

Our study has two major limitations. First, it was a single institution retrospective study and around half of the cases were under 70 years old, not perfectly reflecting elderly patients in a country where the median life expectancy for women is now climbing to 84.8 years [33]. Second, we considered, for statistical analysis, patients who received reduced chemotherapy from the first cycle as no reduction, and this could potentially underestimate the true impact of decreasing dose on survival. There are also some strengths of this analysis. We focused specifically on older patients with cancer, who have been consistently underrepresented in clinical trials. Using data from our practice increases uniformity of treatment and management of patients, as well as replicability of these results in real-world care.

Lastly, we analyzed the correlation between breast cancer and diabetes. Alterations in glucidic metabolism, usually hidden by glucocorticoids use, are common during chemotherapy, involving 10–30% of patients in different cohorts [34]. In our study, 16.8% of the total developed a condition of diabetes, starting specific treatment; these results overtake the ones observed in the Ontario Cancer study [35] that showed an incidence of almost 10% of diabetes after adjuvant chemotherapy, but considering all ages. On the other hand, half of the cases already affected by diabetes had an exacerbation of the disease, increasing the ‘frailty’ of these patients. Close monitoring is recommended in order to control blood glucose level and adherence to therapies [36].

## Conclusion & future perspective

To conclude, cardiologic toxicity affected almost 15% of patients in our over 65-year old population and reduction or discontinuation of adjuvant chemotherapy due to side effects happened in nearly 30%, both potentially worsening outcome. Further work is required to improve patient selection in order to successfully complete adjuvant treatment and minimize side effects, as well as biomarkers able to identify cardiac damage in a very early stage.

Life expectancy is increasing all over the world and consequently so is the proportion of breast cancer diagnoses in elderly patients. First, in our opinion, the major change in the next future should concern clinical trials: till now, elderly patients are often excluded from multicentric randomized trials. This is relevant in clinical practice, since the applicability of new drugs or regimens is not supported by robust data and it is demanded by the oncologist’s empiric attitude. Moreover, the other main challenge is the identification of biomarkers able to accurately predict the development of cardiotoxicity, before the damage will be clinically evident. Lastly, the use of geriatric assessment tools should become routine: the use of high-specific and sensitive items will improve patient selection and stratification in order to reduce side effects and chemotherapy discontinuation.

**Summary points**

- Adjuvant chemotherapy in the elderly is often reduced or interrupted due to a higher rate of side effects, potentially worsening outcome of these patients.
- In our over 65-year old population, dose reduction/discontinuation of adjuvant chemotherapy due to side effects happened in nearly 30%.
- Cardiotoxicity, defined as a reduction greater than 10% in ejection fraction without symptoms or greater than 5% when associated with clinical manifestation, below 55% threshold, happened in 15% of the population.
- Both these events lead to numerically lower progression-free survival, negatively affecting outcomes in this over 65-years cohort.
- Proper selection and closer monitoring of patients could improve tolerance and, therefore, outcome in elderly breast cancer patients.
